# Analysis of MCQ and distractor use in a large first year Health Faculty Foundation Program: assessing the effects of changing from five to four options

**DOI:** 10.1186/s12909-018-1346-4

**Published:** 2018-11-07

**Authors:** Nicolette Fozzard, Andrew Pearson, Eugene du Toit, Helen Naug, William Wen, Ian R. Peak

**Affiliations:** 1 School of Medical Science, Griffith University, Gold Coast Campus, Southport, QLD 4222 Australia; 2 School of Environment and Science, Griffith University, Gold Coast Campus, Southport, QLD 4222 Australia

**Keywords:** Multiple choice question, Discrimination index, Difficulty factor, Reduce options, Distractors

## Abstract

**Background:**

Multiple choice questions are commonly used in summative assessment. It is still common practice for tertiary institutions and accrediting bodies to use five-option single best answer multiple choice questions, despite a substantial body of evidence showing that multiple choice questions with only three or four options provide effective and discriminatory assessment.

**Methods:**

In this study we investigated the distribution of distractor efficacy in exams from four large first-year undergraduate courses in chemistry and in anatomy and physiology in a Health Faculty; assessed the impact on overall student score after changing from five-option to four-option single best answer multiple choice questions; and assessed the impact of changing from five options to four options on item difficulty and discrimination.

**Results:**

For the five-option questions analysed, 19% had four effective distractors, which is higher than previous studies, but still a minority of questions. After changing from five to four options, the overall student performance on all multiple choice questions was slightly lower in the second offering of one course, slightly higher in the second offering of another course, and similar in the second offering for two courses. For a subset of questions that were used in both offerings, there were negligible differences in item difficulty and item discrimination between offerings.

**Conclusions:**

These results provide further evidence that five-option questions are not superior to four-option questions, with reduction to four options making little if any difference to overall performance, particularly when MCQ is used in conjunction with other assessment types (including short answer questions, and practical or laboratory assessment). Further areas of study that arise from these findings are: to investigate the reasons for resistance to changing established assessment practice within institutions and by accrediting bodies; and to analyse student perceptions of the impact of a reduced number of options in MCQ-based assessment.

## Background

Multiple Choice Question (MCQ) assessments provide the advantage of rapid (usually automatic) marking and return of results, which are important considerations for large class sizes requiring rapid turnaround of results.

At Griffith University, students entering a range of undergraduate health programs undertake a foundation year, with common courses in their first two semesters. These courses cater for a large number of students with diverse academic abilities, and many proceed to later postgraduate degrees in health professions. Courses within this foundation program use MCQs to achieve rapid turnaround in marks with very large classes. Many education practitioners use five-option MCQs, that is, one correct and four incorrect (distractor) options, and convention was for foundation year courses to use MCQs with five options. This was in part justified by the fact that some health-related professional bodies use this format of MCQs, including the Australian Medical Council.

There is a substantial body of evidence that MCQs containing only three or four options provide effective and discriminatory assessment [6]. Furthermore, many four- or five-option MCQs suffer from having ineffective distractors, that is, answers that are so implausible that these answers are rarely chosen [2, 7, 11]. Prior studies in nursing and medical programs have examined the effect of reducing MCQ options by modelling effects and redistribution of marks [4, 11]. Other studies examined sequential cohorts: Tarrant and Ware examined a single undergraduate public health nursing course (142 examinees), with some re-used questions [10]. Redmond et al. studied 310 examinees across five courses from second, third, and fourth year of a four-year undergraduate baccalaureate nursing program [5], while Cizek & O’Day studied 700 students in a high-stakes medical specialty exam [1]. What appears to be lacking in published work are studies assessing MCQ changes in large cohorts across multiple courses from different disciplines within health degree programs. In this study, we directly assessed the effects of changing from five-option to four-option MCQs, across four first year courses with large enrolments from diverse health programs (total 5272 examinee responders). We studied two Chemistry and two Anatomy and Physiology courses, assessing changes between sequential year student cohorts. We included a subset of questions in each of the four courses that were used between sequential years, allowing direct comparison of the effect of changing from five- to four-option questions.

## Methods

### Data collection

Item analysis data was retrieved for two first year anatomy and physiology courses (designated here as A&P A and A&P B) offered in semester 1 and semester 2, respectively, as well as two first year chemistry courses (here, Chem A and Chem B), similarly offered in semester 1 and semester 2, respectively, at Griffith University (Queensland, Australia). Students must pass A&P A and Chem A before they can undertake A&P B and Chem B, respectively.

The item analysis data was calculated and supplied in reports generated by examSYSTEM II software (Scantron, Minnesota, USA) and included discrimination index for each question option (response-specific), and difficulty factor for each question (question-specific). Discrimination index (DI) measures the extent to which a particular item response (distractor or correct option) is able to discriminate between individuals who attain a high score on the overall MCQ result (across all MCQs) and those that attain a low score; specifically for these data, *DI = (U-L)÷N*_*U*_, where *U* is the number of students in the upper quartile that selected that response, *L* is the number in the lower quartile, and *N*_*U*_ is the total number of people in the upper quartile. Correct responses tend to have positive DI, while distractors are negative, and the closer the DI to one (1) or negative one (− 1), respectively, the more discriminatory a response is. Difficulty factor (DF) is the proportion of respondents that select the correct option out of the total number of respondents for that question. Other useful data used from the item analysis was the % of respondents that each distractor elicited. Distractors were classed as ineffective distractors if ≤5% of respondents chose that answer, in line with criteria suggested or previously used [2, 10].

Analysis involved considering data at two time points: the first offering when five-option MCQs were used, and in the following year (second offering) when four-option MCQs were used. All questions in the second offering had four options. To directly compare the effect of reducing from five to four options, a subset of questions for each course were identical between offerings, with the only difference being the removal of the least effective distractor for the second offering. The number of questions re-used, and which questions, was at the discretion of individual course convenors.

Anonymised student demographic data were acquired from the University’s Planning and Statistics databases.

### Data analysis

To investigate if changing the number of distractors affected the overall mark distribution, independent *t*-tests were used to assess the differences in both overall MCQ score, and differences in student outcomes for the subset of questions that were repeated in two offerings of the course (first offering: five-option questions; second offering: four-option questions). Independent *t*-tests were most appropriate since the cohorts of students across years were considered independent of each other, with largely unique students in each sample. Unequal variances independent *t*-tests were used where Levene’s test showed significant differences in variances. These were performed for each of the four courses.

To investigate the effect of the change in difficulty and discriminatory power by removal of the least effective distractor, paired *t*-test analysis was performed for each of these measures, for each course. Paired *t*-test analyses were used due to the repeated use (and therefore similarity and relatedness) of the questions and responses; thereby, the item analysis measures (DI and DF) could not therefore, be considered independent. The questions used in these analyses formed a subset of the overall MCQ section of the exam. Additionally, these measures for all courses were pooled and analysed using a 4 × 2 mixed model ANOVA where course and offering, respectively, were the main effects investigated for each measure of DF and DI. The interaction between course and offering was not significant (for DI, *p* = .533; DF, *p* = .10). This multivariate, pooled approach allowed for an overall analysis, with larger sample size, and enabled the pre-post comparison of each of DF and DI, while considering the course from which the data was drawn.

Normality assumption testing involved the use of Q-Q plots, frequency histograms (with normal curve overlaid) and Shapiro-Wilks Test of Normality. This testing found that Normality was met for all analyses.

All analyses were conducted as two-tailed, with *p* = .05 used as the threshold for statistical significance, using SPSS Statistics for Windows, Version 24 (IBM Corp.). Graphs of the relationship between DF and DI, and changes in DF and DI, were generated in Microsoft Excel 2013.

## Results

### Description of cohort

**Fig. 1:**
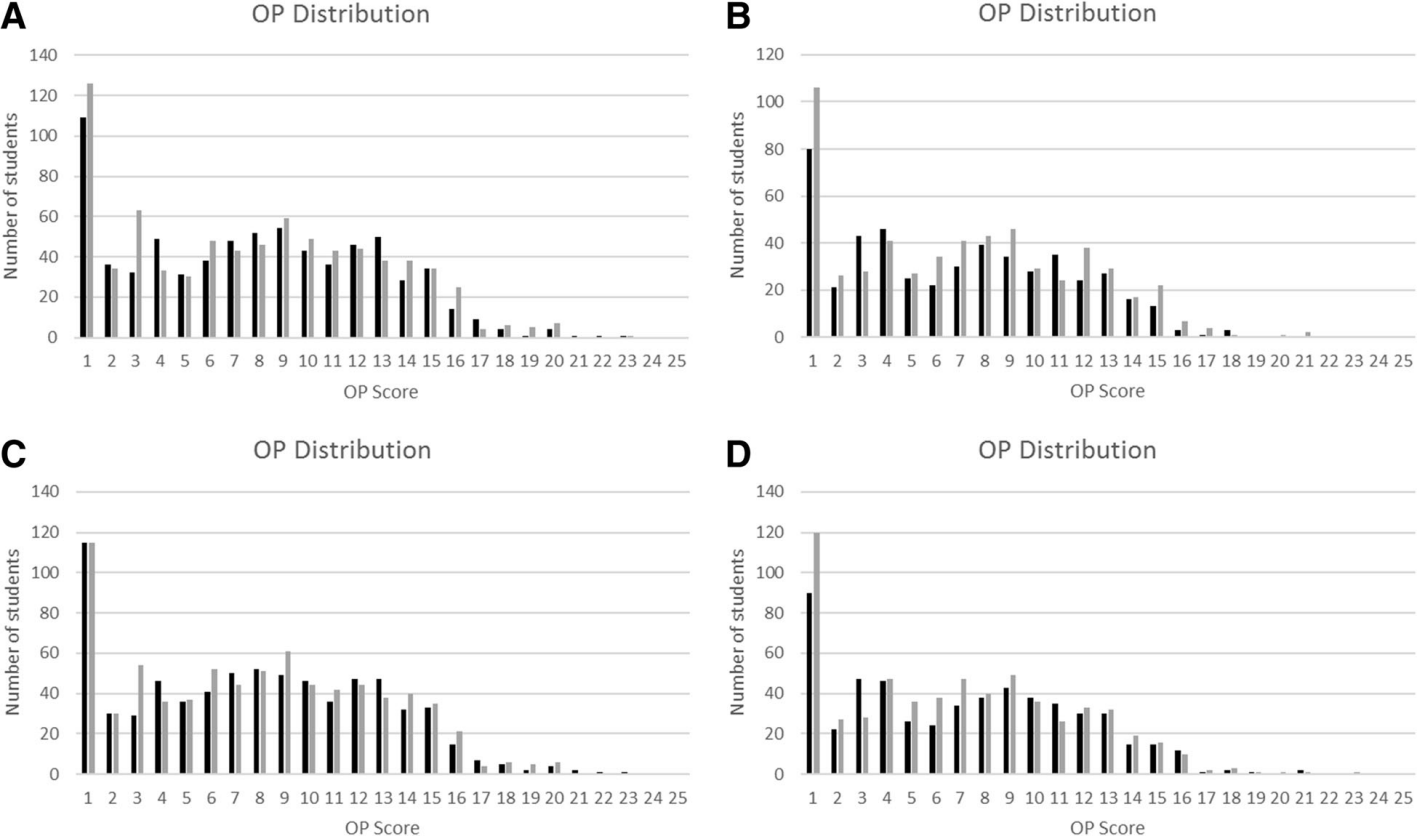
Comparison of student entry scores. Entrants’ high school academic scores (OP) in Chem A (**a**), Chem B (**b**), A&P A (**c**), and A&P B (**d**). First and second offerings are indicated by black and grey bars respectively

At the Griffith University Gold Coast campus, students entering a range of undergraduate health-related degree programs undertake common courses in their first year, known as Foundation Year. Degrees utilising these common courses include Health Science, Biomedical Science, Medical Science, Exercise Science, Pharmacy (and related Programs), Dental Science, Nutrition and Dietetics, Medical Laboratory Science, Environmental Health, and Public Health. The common Foundation Year courses were studied in a two-semester academic year, each of 13 weeks of teaching. These courses cater for a large number of students with diverse academic abilities with the average student scores for Foundation Year Programs in 2015 ranging from 1.0 to 13.2 (where high-school graduates are ranked on a bell curve from 1 to 25, called an Overall Position or OP). The distribution of entrant scores was not different between two years for each of the courses examined in this study, but the distributions highlight that there are several programs requiring high entry scores (OP 1), compared with other programs (Fig. 1). In addition, programs had differing requirements for prior high-school study of sciences, with some requiring multiple science and advanced mathematics pre-requisite knowledge, and others requiring only completion of English and one of maths, biology, chemistry or physics.

Included in the common Foundation Year are two sequential anatomy and physiology courses and two sequential chemistry courses. Students must pass Chem A before attempting Chem B, and must pass A&P A before attempting A&P B. Student numbers in these courses ranged from 512 to 770 students (Table 1). The courses are assessed by a range of methods, but large class size and requirement for rapid turnaround of student marking necessitates the inclusion of MCQs as a major element of the assessment (Table 2), being up to 50% of the final exam. All courses also have a laboratory class component (20–25% weighting per course) which is assessed by a variety of methods, including quizzes with short response and/or MCQs, workbooks, reports, and competency tests. Prior to this study, the convention for Foundation Year exams was for MCQs to have five options, i.e. one correct answer and four distractors.

**Table 1:** Demographic data of students

	Chem A		Chem B		A&P A		A&P B	
	1st offering	2nd offering	1st offering	2nd offering	1st offering	2nd offering	1st offering	2nd offering
Number of students	741	767	512	551	736	770	581	614
% Female	53	52	54	52	53	53	54	55
Ave age (start of year)	20.1	19.6	19.4	19.9	19.7	19.3	19.5	19.9
% age 20+	27	25	25	28	25	23	25	27
% ESL^a^	22	22	21	24	23	22	21	26
Avg OP^b^	7.82	7.75	6.86	7.08	7.86	7.81	7.11	6.92
% SES^c^ (High/Med/Low)	15/65/11	14/63/14	15/59/11	16/65/11	15/66/11	13/65/13	14/60/12	17/64/11
% ATSI^d^	2	2	1	2	2	2	1	2

^a^English as a Second Language

^b^OP is school leaver qualification (scale of 1 to 25, with 1 being the highest achievement)

^c^Socioeconomic status; may not add up to 100% due to rounding and/or unavailable data

^d^Students self-identifying as Aboriginal and Torres Strait Islander

**Table 2:** Summary of final exam MCQ assessment

Course	Weighting assigned to final exam	# MCQs in final exam	% of final exam assessed by MCQ
Chem A	55%	25	50
Chem B	55%	50	50
A&P A	50%	60	44
A&P B	50%	60	50

For each course, student demographics were similar across corresponding semesters (S2 2013 to S2 2014, and S1 2014 to S1 2015): average age at the start of year was 19.3–20.1 years, 23–28% were aged over 20 years of age; gender, 52–55% females, 45–48% males; English as Second Language, 21–26%; average OP score, 6.86–7.86; socioeconomic status, 13–17% high, 59–66% medium, 11–14% low; and Indigenous students, 1–2%. (Table 1).

### Distractor analysis at first offering

To determine how effective the distractor answers were, responses to end-of-semester exam MCQs were analysed from the first offering. Distractors were regarded as effective if more than 5% of students chose a response. This showed that courses had variable number of questions with four effective distractors ranging from 4 to 28% (Table 3). In our study, 19% of the five-option questions (*n* = 195 across all four courses) had four effective distractors. The most frequent number of effective distractors per question was three, with 32% of the 195 questions. Overall, 7% of questions had no effective distractors. The result of this analysis showed only a minority of questions had four effective distractors. This is consistent with other studies that describe distractor effectiveness (see for example [11]). In the second offering, the number of distractors was reduced to three for all questions. We assessed the effect of this change on overall student performance, and then analysed the psychometric measures of a subset of questions that were used in both first and second offerings.

**Table 3:** Percentage of questions with specified number of effective distractors at first offering

Course^a^	Number of Effective Distractors (> 5% of respondents)				
	Four	Three	Two	One	None
Chem A (*n* = 25)	4%	32%	24%	24%	16%
Chem B (*n* = 50)	22%	32%	20%	18%	8%
A&P A (*n* = 60)	13%	42%	33%	8%	3%
A&P B (*n* = 60)	28%	23%	27%	17%	5%

^a^*n* denotes the total number of MCQs used in the final exam and is the denominator for percentages shown

Percentages may not add up to 100% due to rounding

### Analysis of student performance after the change from five to four options

The overall student performance on all MCQs was compared between first and second offering (Table 4). For the Chem A exam, the student scores were slightly lower at second offering (by 0.8 marks out of 25), and in A&P A overall scores were slightly higher (by 1.3 marks out of 60). In the other two courses, marks were similar between offerings, with no statistically significant difference between the cohorts (*p* > .05).

**Table 4:** Overall score in MCQ in the two offerings

Course^a^	No. of students sitting exam		Results^b^		*p-*value
	1st offering	2nd offering	1st offering	2nd offering	
Chem A (*n* = 25)	741	767	19.0 ± 4.37 (76.2%)	18.2 ± 4.47 (72.9%)	<.001
Chem B (*n* = 50)	512	551	33.8 ± 9.11 (67.6%)	33.3 ± 8.25 (66.6%)	.336
A&P A (*n* = 60)	736	770	40.1 ± 10.77 (66.8%)	41.4 ± 9.30 (69.0%)	.011
A&P B (*n* = 60)	581	614	39.2 ± 10.00 (65.4%)	39.6 ± 10.13 (66.0%)	.538

^a^*n* = total number of MCQs used in the final exam and is the denominator for percentages shown. In 1st offering, all questions are five-option, in second offering all questions are four-option

bMean mark ±1 SD; average % mark given in parentheses

**Fig. 2:**
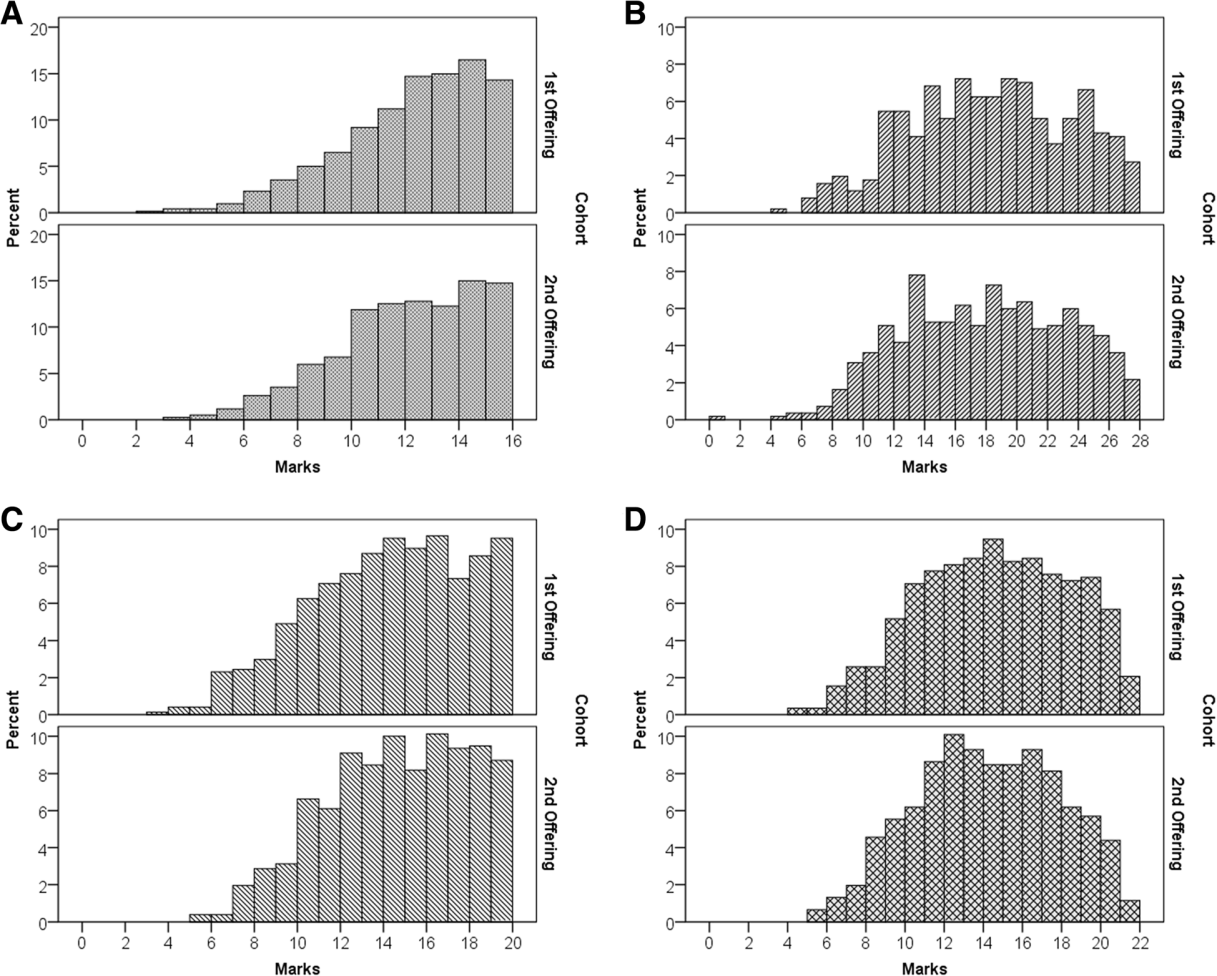
Distribution of marks achieved in questions that were kept the same between sequential academic years. First offering had 4 distractors, second offering had 3 distractors. Mark distributions were similar across all courses. Panels: (**a**) Chem A; (**b**) Chem B; (**c**) A&P A; (**d**) A&P B

A more granular view was provided when we compared the subset of questions that were re-used between years. These questions only differed in the second offering by having the least effective distractor removed. Distributions were analysed using an independent samples *t*-test (Fig. 2). Three out of the four courses showed no significant difference between offerings (*p* > .05, Table 5); that is, removal of the least effective distractor resulted in no significant change in student performance from first to second offering on those questions in three courses. In A&P A, the percent score on the repeated questions increased from 69.7 to 72.2% (*p* < .01).

**Table 5:** Comparison of MCQ marks for re-used questions

Course^a^	No. of students sitting exam		Results^b^		*p*-value
	1st offering	2nd offering	1st offering	2nd offering	
Chem A (18/25 questions)	741	767	11.8 ± 2.6 (78.4%)	11.6 ± 2.6 (76.6%)	.188
Chem B (28/50 questions)	512	551	17.8 ± 5.2 (65.8%)	17.5 ± 5.3 (64.6%)	.333
A&P A (20/60 questions)	736	770	13.9 ± 3.7 (69.7%)	14.5 ± 3.5 (72.2%)^%^	.007
A&P B (24/60 questions)	581	614	14.1 ± 3.8 (67.1%)	13.7 ± 3.6 (65.4%)	.116

^a^(number of questions repeated in both offerings/total number of MCQ in the exam)

^b^Mean mark ±1 SD; average % mark on these re-used questions given in parentheses

### Effect on difficulty and discrimination

The difficulty and discrimination of the repeated questions were analysed. When the DI and DF data from questions from all four courses were pooled and analysed by multifactorial mixed model ANOVA (Table 6) there was no significant difference in DI (*p* = .26) and DF (*p* = .58) either for the main effect of offering, or between-subjects effect of the courses (*p* = .33 and .09, respectively). However, in one course (A&P A), a small difference was apparent in DF, with an average decrease in question difficulty by .025 (95% CI: .007 to .043). The change in DI was not significant (mean = −.040; 95% CI: −.084 to .004). These results suggest that the four-option offering was slightly “easier” in only the A&P A exam. The change in difficulty for the A&P A exam (Table 6) was also consistent with the slight increase in scores for the second offering of re-used questions in the A&P A course (Table 5).

**Table 6:** Question difficulty and discrimination with four and three distractors

Course	First offering four distractors		Second offering three distractors		Average Difference⋄ (*p*-value)	
	DF^b^	DI^b^	DF^b^	DI^b^	DF	DI
Chem A (*n* = 15)^a^	.78 ± .14	.41 ± .16	.77 ± .14	.41 ± .18	−.012 (.06)	.01 (.67)
Chem B (*n* = 27)^a^	.66 ± .17	.49 ± .17	.65 ± .16	.49 ± .14	−.012 (.45)	−.00 (.88)
A&P A (*n* = 20)^a^	.70 ± .17	.46 ± .19	.72 ± .17	.42 ± .16	.025 (.01)	−.04 (.07)
A&P B (*n* = 21)^aa^	.67 ± .18	.43 ± .15	.65 ± .17	.41 ± .13	−.016 (.27)	−.01 (.53)
All courses (*n* = 83)	.69 ± .17	.45 ± .17	.69 ± .17	.44 ± .15	−.004 (.56)	−.01 (.24)

^a^*n* denotes the number of questions used in first and second offerings, where the least effective distractor was removed at second offering

^b^Mean ± 1 SD

⋄Average difference: the difference in DF and DI between offerings was calculated for each question (second offering minus first offering), and the average change calculated for each course; *p*-value shown in parentheses

To investigate the effect of removing the least effective distractor on questions that already had four effective distractors, we examined the subset of 15 repeated questions that had four effective distractors in the first offering. The difficulty factor increased on average by 0.01 (95% CI: -0.04 to 0.06), and the discrimination index reduced on average by 0.09 (95% CI: -0.14 to − 0.05).

**Fig. 3:**
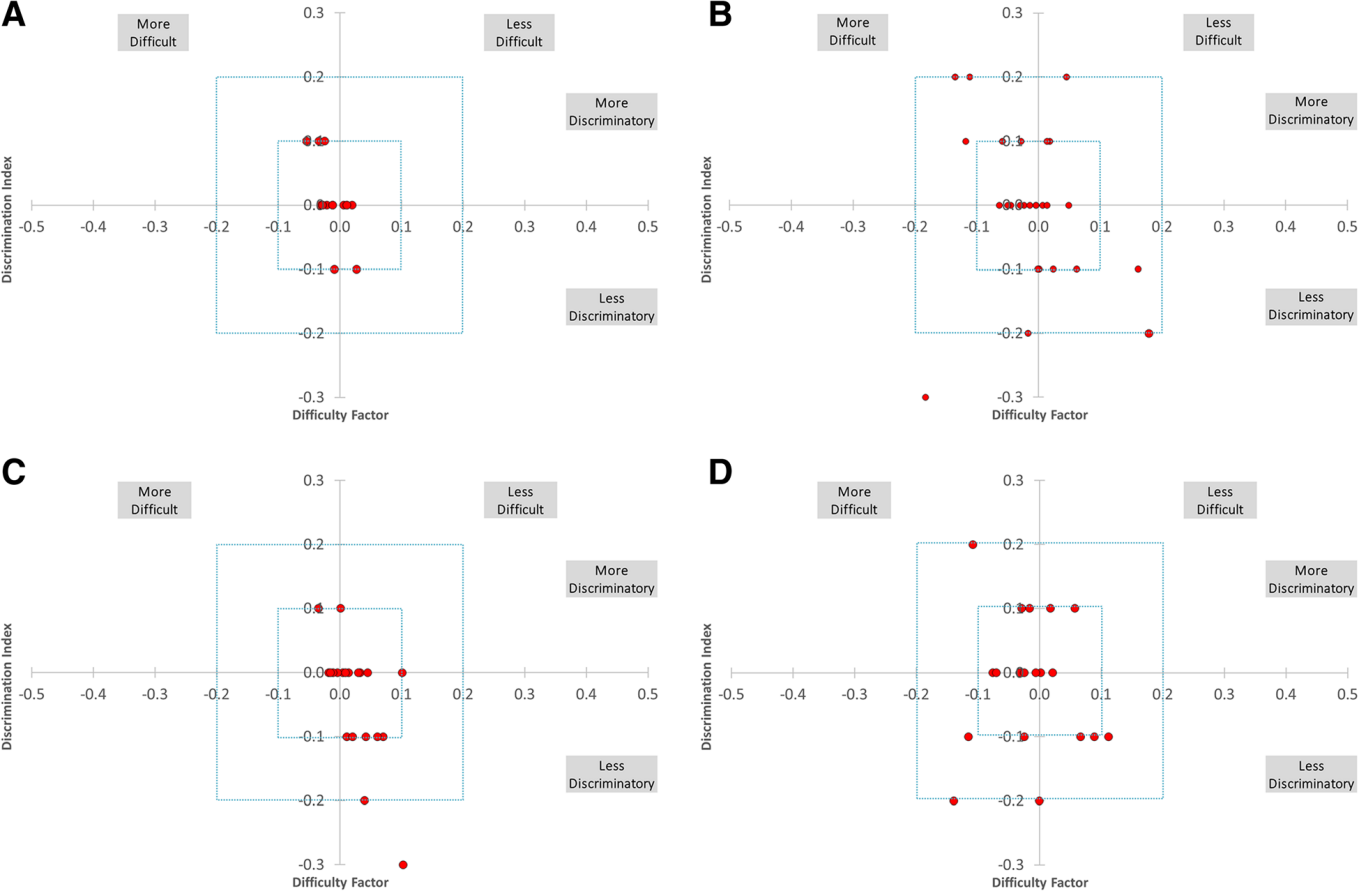
Change in discrimination index and difficulty for individual questions after removing least effective distractor. Most questions showed minimal changes of ≤.1 units in DF and DI. Panels: (**a**) Chem A (*n* = 15); (**b**) Chem B (*n* = 27); (**c**) A&P A (*n* = 20); (**d**) A&P B (*n* = 21)

Another assessment of the effect on individual questions was made by plotting the change in difficulty and discrimination for each question (Fig. 3). This showed that the DF and DI of most questions changed by ≤0.1.

**Fig. 4:**
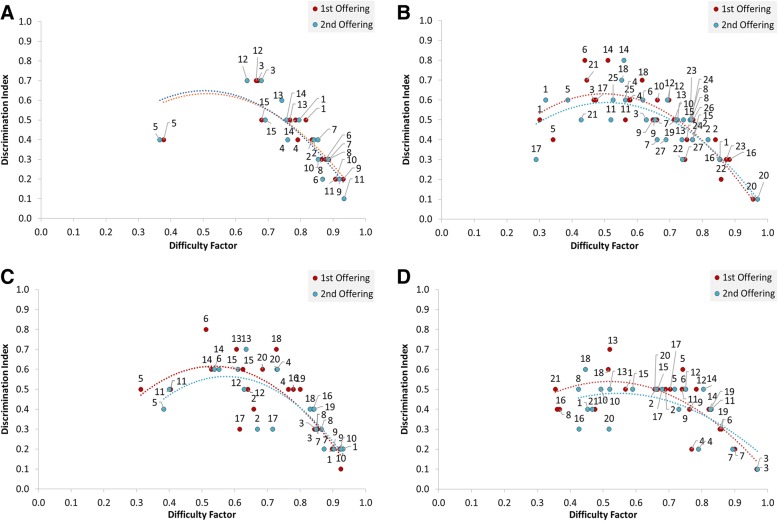
Relationship between item difficulty and discrimination before and after removal of least effective distractor. Numbers indicate the question number in each of the offerings (label above respective marker, or indicated by leader lines). Second order polynomial trend lines (without pre-specified intercept) added to each offering dataset. Panels: (**a**) Chem A; (**b**) Chem B; (**c**) A&P A; (**d**) A&P B

To visualise the difference in DF and DI, values were plotted for each course, comparing the subset of questions between offerings (Fig. 4). The relationship between DF and DI is not linear, but describes a dome. This is consistent with previously reported analyses of this relationship (see for example [3, 8]. The trendlines (second-order polynomial, as fit by Microsoft Excel) for each course were similar between offerings, showing that four-option MCQs did not significantly affect this relationship between DF and DI.

## Discussion

Our study examined large cohort first year courses in chemistry and anatomy & physiology, with up to 770 examinees in a course. Assessment convention was for five-option questions (i.e.*,* four distractors). Students from the courses in this study were enrolled in undergraduate programs that feed into medical and health professions including medicine, dentistry, pharmacy, and physiotherapy. In Australian health professions, accrediting bodies use MCQ examinations. The number of options in these MCQs varies, with the Australian Medical Council and the Australian Dental Council using five option MCQs, while the Pharmacy Council MCQs are “four or five options”, and the Physiotherapy Council uses four-option MCQs.

It is recognised that it is not trivial to generate a large bank of questions with four effective distractors [11]. In our study, we first examined the effectiveness of the distractors in our five-option questions. Of the five-option questions, 19% had four effective distractors and 32% had three effective distractors. This is higher than previously reported studies of health care courses: Rogausch et al. found only 2.8% of five-option questions in a Swiss Federal medical graduation exam had four effective distractors, and Tarrant & Mohammed found 13.8% of four-option questions had three effective distractors across a number of nursing courses [7, 11].

We evaluated the effect of reducing from five- to four-option questions in sequential years. The use of sequential years was advantageous as the two independent cohorts decreased the threat of single-group (for example, giving the same students a set of questions twice, the second with the least effective distractor removed) testing bias, where students can be “primed” by learning from earlier exposure to materials/questions or providing a subsequent opportunity at correctly answering the questions. However, as these courses are core (required) components of the degree programs, failing students are required to retake the course the following year. Thus, a proportion of each second offering cohort will be repeating students. These students, and their re-seeing the same questions may be considered a potential confounding effect. However, at least one study has shown that repeating examinees tend to pick the same answer at their second attempt, and not from remembering the question [13]. We therefore discounted the effect of repeating students on psychometric measures.

To address concerns that reducing the number of options might make the exam easier by increasing the probability of guessing the correct answer (i.e., reduce difficulty), a subset of questions used on both offerings was analysed. For the second offering, the least effective distractor was removed (as determined by analysis of responses to questions from first offering). A previous study has suggested that the method of removing distractor has limited effect on DI or DF [6]. It should be mentioned that this is a series of foundational courses aimed at providing all students with a baseline knowledge, so some questions are included to assess threshold knowledge, hence there are questions that almost all students obtain the correct answer (0, or 1 effective distractor). Overall, 7% of questions had no effective distractors. This is lower than reported from other studies of healthcare education in which 14.2% of questions evaluated in a UK medical school [4], and 12.3% of questions from courses in a Hong Kong nursing school [11] had no effective distractors.

Assessing a subset of re-used questions, we found there were no or slight changes in DF or DI between offerings. This is consistent with a previous meta-analysis, in which “Moving from 5-option items to 4-option items reduces item difficulty by .02, reduces item discrimination by .04” [6]. Other authors reviewed literature and found no differences in psychometric properties of three-option tests when compared with 4 and 5 options [12]. Individual studies also confirm minimal changes in psychometric properties when reducing number of options, either in a theoretical redistribution of marks [4, 7], or in testing in sequential academic year cohorts ([10]; Cizek and O’Day; [5]) as was the case in the current study.

Concerns expressed by staff about reducing the number of options in MCQs were that removing a distractor may make the exam easier (i.e., increased marks through guessing), or that discrimination of the questions is reduced. Interestingly, this concern is reportedly shared by students, who felt that reducing options would be less fair as it would make exams easier [4]. Our results corroborate other studies that suggest this fear is unfounded. Indeed, one author in a meta-analysis suggests that reducing options does not lead to increased correct answers by guessing; even in three-option questions guessing is unlikely because “Examinees are unlikely to engage in blind guessing, but rather educated guessing where the least plausible distractors are eliminated, essentially reducing the 4- or 5-option item to a 3- or 2-option item” [6]. However, for the worst performing students, it is not clear whether their knowledge is sufficient even to assess what is the least plausible, that is*,* whether, as Kilgour and Tayyaba assume, students who pick the least effective distractor are indeed guessing [4]. This is an ongoing area of study within our large cohort first year courses that are taken by students with a wide range of starting academic capital and knowledge. Nevertheless, the evidence we present in these large cohort classes is consistent with most other literature that shows reducing from five options to four has negligible impact on performance in MCQs.

The removal of the least effective distractor is an important strategy in reducing the number of distractors, while maintaining the quality of the MCQ. The small potential effects on marks or discrimination are outweighed by the benefits found in reducing options. These include reduced time to answer questions [9, 12] with increased potential to cover more content in the same time [6], as well as reduced burden on question writers to script additional distractors. For students who speak English as a second language (in our cohorts, around one-quarter of the students), fewer distractors requires less time and decoding of the options.

Despite the evidence that five-option questions are not superior to four-option MCQs (which our study reiterates and corroborates), there is still some resistance from some stakeholders at our institution to reduce the number of options in MCQ assessment. The basis for this resistance is unclear, despite evidence of no effect on difficulty or item discrimination, and may be an area for future research. Further reduction to three-option MCQs is of interest using the quasi-experimental methods employed here.

In these courses, no more than 50% of student learning was assessed using MCQ in the final exam. Therefore, even if the small difference seen in difficulty and discrimination for A&P A is extrapolated to other courses, the breadth of assessment types result in little overall difference in most students’ performance in the courses.

## Conclusions

These results are consistent with prior reports from health-related education and other disciplines in that few MCQs have all-effective distractors. Our data provide evidence in a large foundation year cohort across different heath disciplines that reducing option number from five to four has negligible impact on question difficulty, student marks, or discrimination power of questions.

## Abbreviations

DF: Difficulty factor
DI: Discrimination Index
MCQ: multiple choice question
